# Structural Biology and Molecular Modeling to Analyze the Entry of Bacterial Toxins and Virulence Factors into Host Cells

**DOI:** 10.3390/toxins11060369

**Published:** 2019-06-24

**Authors:** Irène Pitard, Thérèse E Malliavin

**Affiliations:** 1 Unité de Bioinformatique Structurale, Institut Pasteur and CNRS UMR3528, 75015 Paris, France; irene.pitard@pasteur.fr; 2 Centre de Bioinformatique, Biostatistique et Biologie Intégrative, Institut Pasteur and CNRS USR3756, 75015 Paris, France; 3 Sorbonne Université, Collège Doctoral, Ecole Doctorale Complexité du Vivant, 75005 Paris, France

**Keywords:** anthrax, botulinium toxin, diphtheria toxin, bordetella pertussis, adenylyl cyclase, molecular modeling, enhanced sampling, drug design, calmodulin

## Abstract

Understanding the functions and mechanisms of biological systems is an outstanding challenge. One way to overcome it is to combine together several approaches such as molecular modeling and experimental structural biology techniques. Indeed, the interplay between structural and dynamical properties of the system is crucial to unravel the function of molecular machinery’s. In this review, we focus on how molecular simulations along with structural information can aid in interpreting biological data. Here, we examine two different cases: (i) the endosomal translocation toxins (diphtheria, tetanus, botulinum toxins) and (ii) the activation of adenylyl cyclase inside the cytoplasm (edema factor, CyA, ExoY).

## 1. Introduction

Structural biology evolved from the determination of the structure of small metalloproteins [1] that are moderately flexible around a stable conformation up, carried out in the late 50s, to the current studies of intrinsically disordered proteins [2]. Such development has been accelerated by the expansion of biophysical techniques. The combined uses of these methods provides an increasing amount of information on structure and dynamics of biomolecular systems. One major result of this development is the recognition that structure, dynamics and conformational transitions are intricately connected in all biomolecular systems, and they are also closely related to the cellular and physiological processes in which the biomolecules are involved. In addition, due to intrinsic gaps in experimental studies, the parallel use of molecular modeling and structural biology approaches is essential to understand protein function, in particular in the case of conformational flexibility. Indeed, transient phenomena as mobility or conformational transitions are difficult to experimentally quantify, as most experimental techniques used in structural biology only gain sufficient sensitivity by time and/or space averaging of the signal.

Molecular modeling approaches face other issues. One long-lasting problem is the size of the conformational space that must be explored, which is still a challenge despite the development of high-power computing and of enhanced sampling techniques [3,4,5]. Another problem is the limitations induced by the physical model of inter-atomic interactions or *force-field* [6,7]. Nevertheless, in numerous examples, the use of molecular modeling along with structural biology or biophysics approaches allowed a deeper investigation of the system [8,9,10].

In the present review, we present how the cooperative use of in silico and experimental structural biology approaches improve our knowledge of specific steps of the insertion of bacterial toxins and virulence factors into the host cells. We focus the review on two events, widely recognized as essential in the toxicology field: (i) the translocation of diphtheria, botulinic and tetanus toxins through the membrane of endosome vesicles, (ii) the activation of adenylyl cyclases in the cytoplasm of host cells. The choice of these biological topics was motivated by the existence of several X-ray crystallographic structures of these toxins, as well as by the presence of large conformational transitions investigated by molecular modeling studies.

## 2. Bacterial Toxins

### 2.1. Diphtheria Toxin

**Figure 1:**
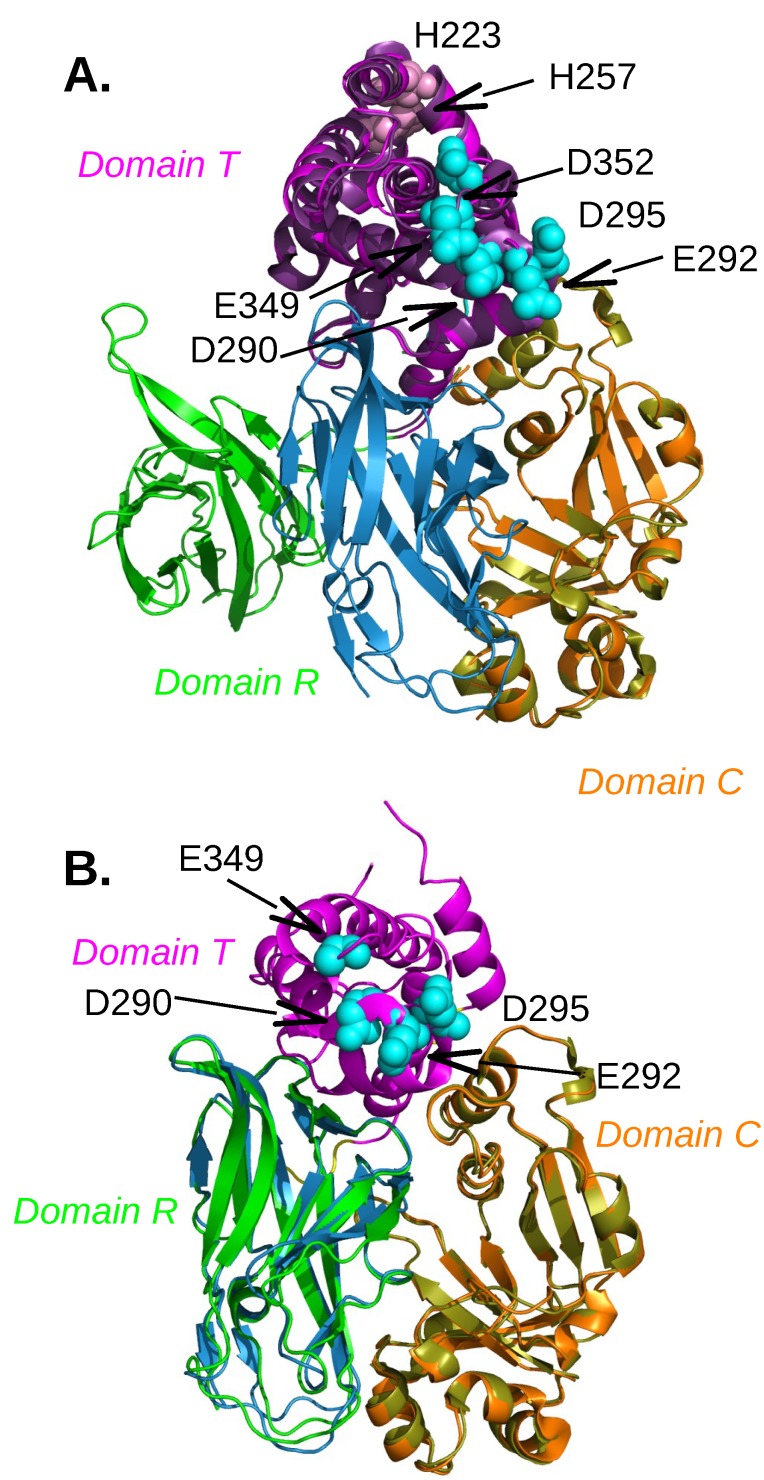
Diphtheria toxin (DT) structures determined with closed conformation (PDB entry: 1F0L [18]), with open conformation (PDB entry: 1DDT [12]), at acidic pH (PDB entry: 4OW6 [17]). The structures are drawn in cartoon. (**A**) Superimposition of the structures 1DDT and 1F0L on the domain C, The domains are colored in the following way: domain C (orange for 1DDT, olive green for 1F0L), domain T (magenta for 1DDT, violet for 1F0L), domain R (green for 1DDT, turquoise for 1F0L). The moving apart of the green domain T in the open conformation of 1F0L is visible. The residues D290, E292, D295, E349, D352, drawn with spheres and colored in cyan, are located in the dagger tip. The residues H223 and H257 are drawn as spheres and are colored in pink. (**B**) Superimposition of the structures 4OW6 and 1F0L on the domain C, The domains are colored in the following way: domain C (orange for 4OW6, olive green for 1F0L), domain T (magenta for 4OW6, not shown for sake of clarity for 1F0L), domain R (green for 4OW6, turquoise for 1F0L). The residues D290, E292, D295, D352, drawn with spheres and colored in cyan, are located in the dagger tip. The region containing residues H223 and H257 is unfolded in 4OW6 because of the acidic pH conditions.

Diphtheria toxin (DT) was recognized as a major disease-causing agent at the end of 19th century, when filtered bacterial culture was shown to reproduce the disease in animal models. This 58 kDa protein includes three domains: the catalytic (C) domain (residues 1–193), the middle translocation (T) domain (residues 205–378) and the receptor-binding (R) domain (residues 386–535) (Figure 1). The monomeric DT [11] is a Y-shaped molecule, formed by two fragments A and B connected by a disulfide bridge. The fragment A corresponds to the domain C, which contains α and β secondary structures, colored in orange or in olive green in Figure 1. The fragment B contains: (i) the domain T, formed by 9 α helices labeled TH1 to TH9, and colored in magenta or in purple in Figure 1; (ii) the domain R, a flattened β barrel, colored in green or in turquoise in Figure 1 and displaying an overall topology similar to those of the immunoglobulin (Ig) domains [11].

DT is endocytosed into the cells by binding its fragment B to the heparin-binding epidermal growth factor-like precursor (HB-EGF). Under acidic conditions of the late endosome, the domain C is cleaved and translocated into the cytosol. Once in the cytoplasm the C domain then catalyzes the transfer of the ADP-ribose moiety of NAD onto the elongation factor-2 (EF-2), rendering EF-2 inactive and consequently causing protein synthesis to stop in the host cell.

Several structures of DT [11,12,13,14,15] revealed either monomeric or dimeric forms, each containing its own arrangement of the three domains. The monomeric DT displays a packed arrangement of the three domains, whereas the dimeric DT is formed by swapping of the R domains from two monomeric DT [13] in extended conformation (Figure 1A). Dimerization of DT can be induced by freezing the protein in mixed phosphate buffers, which are known to decrease in pH from 7.0 to 3.6 during freezing. This observation led Carroll et al. [16] to propose that the decrease in pH causes dimerization and, on the basis of this study, it has also been proposed [13] that lowering the pH can convert the monomeric DT into an open form. The pH effect is supported by the existence of numerous charged and polar residues present at the interface between R and C domains. Indeed, the three salt bridges stabilizing the interface at neutral pH will be disrupted at low pH leading to the transition toward the extended monomer structure [13]. Within the T domain, the hydrophobic helices TH8 and TH9 are sandwiched by two layers of amphiphilic helices, TH1 to TH4 and TH5 to TH7. The loops between TH5 and TH6-7 and between TH8 and TH9, which contain acidic residues D290, D292, D295 and E349, D352, respectively, are called the dagger tips [12] (Figure 1A). At acidic pH, these acidic residues can be protonated, thus changing their solubility preference from water to membrane. This hypothesis is confirmed by an X-ray crystallographic structure of DT at low pH [17] (Figure 1B), which reveals an unfolding of the TH2, TH3 and TH4 α-helices from the domain T which exposes an hydrophobic surface that includes the TH5 and TH8 α-helices, and the loop region connecting the TH8 and TH9 α-helices.

The membrane translocation and the effect of acidic pH were further studied by a large set of biophysical methods: fluorescence [19,20,21,22,23], specular neutron reflectometry and solid-state nuclear magnetic resonance (NMR) spectroscopy [24], substituted-cysteine accessibility [25], X-ray crystallography [17], hydrogen-deuterium exchange coupled to mass spectrometry (HDX-MS) [26,27], site-directed spin labeling [28], site-directed mutagenesis [29,30], hydrophobic photo-labeling [31], and introduction of disulfide bridges [32]. These studies provide a very contrasting overview of the conformational transition of DT at low pH and of its translocation through the membrane.

Using fluorescence techniques, several pieces of information were obtained on the behavior of the T domain within DT: (i) the hydrophobic helices TH5-TH9 tend to be more exposed to aqueous solution in the isolated T domain [20]; (ii) the dagger tip connecting TH5 to TH6-7 is stable on the cis (insertion) side of the membrane in the isolated T domain, whereas it moves between the cis and trans sides of the membrane if the domain C is present [20]; (iii) the TH6-TH7 segment displays [25] constriction in the channel possibly formed by the T domain in the membrane; (iv) the T domain acts as a chaperone for the C domain translocation at acidic pH [21]; (v) the mutations E362Q and E349D/D352N, located in the region TH8–TH9 (named as the dagger tip), cause changes to how T inserts into the membrane and the E362Q mutation induces insertion of TH8-TH9 in the membrane at neutral pH [22]; (vi) the protonation of the residue H257, located in the T domain, acts as a pH-sensitive switch that triggers conformational change, resulting in T insertion into the membrane, and the neighboring residue H223 was suggested as a modulator (safety-latch) of H257 protonation [29]; (vii) the H223Q mutant, which lacks the latch, displays unfolding at less acidic conditions (pH < 7.5) with respect to the pH < 6.5 required for WT protein unfolding [30].

The effect of pH was also studied on the isolated T domain. Using hydrogen/deuterium exchange experiments coupled to mass spectrometry [26], the structure of the T domain isolated at neutral pH displays high protection for α helix TH5, which is assigned to the formation of dimers. However, at acidic pH the molten globule state displays high protection within the helical hairpin TH8-TH9, which was assigned to self-assembly of this hydrophobic part of the domain, due to oligomerization. Nevertheless, pH-dependent HDX [27] kinetic analysis reveals a different picture in which transition occurs from the native state (W-state) to a membrane-competent state (W+-state), with exposure to the solvent of the hairpin TH8-9.

Neutron reflectivity experiments [24] provided direct evidence that the T domain’s C-terminal helices penetrate deeply into the hydrocarbon core, while its N-terminal helices penetrate the polar head-group region. This penetration is performed via a pH-dependent, two-step process: (a) a destabilization of the native state through the protonation of histidine side chains (pH 7 to 6) followed by the interaction between the solvent-exposed hydrophobic surfaces of the molten-globule state and the membrane (pH 7 to 6); (b) the reorganization of the T domain conformation leading to a membrane-inserted state (pH 6 to 4). Cysteine-scanning mutagenesis performed [28] on 28 consecutive sites comprising the TH8 helix and the TH8-TH9 inter-helical loop, reveals upon membrane binding at pH 4.6, a major structural reorganization of the domain, with most of TH8 retaining its helicity and the TH8-TH9 loop converted into a new helical structure. Among double mutant cysteines, all mutants forming internal disulfide bridges within the catalytic domain were translocated less efficiently [32].

Several results have been reported for the oligomeric states of DT and of the domain DT in the membrane. It was hypothesized [31,33] that DT exists as an oligomer in membranes. A concerted approach including fluorescence quenching with molecular dynamics (MD) simulations [23] proposes an open-channel model. It was also proposed that there is no single conformation in the transmembrane state, but, rather, a collection of states with different folds and topologies [34]. It was also observed that in the channel formed by an isolated T domain in the membrane contains only one T domain molecule [35]. Besides, it was established [36,37] that two conformations of the T domain can exist in the membrane with helices TH5-9 close to the membrane surface (P state) or more deeply inserted (TM state). The loop between TH8 and TH9, corresponding to one dagger tip, was exposed to the trans side of the bilayer, while other solvent-exposed residues in the helices TH5-9 region are located near the cis surface [19].

To investigate the extensive but contradictory experimental information on the T and DT interaction with the membrane, several molecular modeling studies have been realized. Flores et al. [38] developed an original accelerated MD (DISEI-aMD) approach that biased the electrostatic interactions between atom pairs of the solute: the corresponding MD trajectories showed that, in agreement to circular dichroism and fluorescence experiments, the protonation of histidines triggers partial unfolding of N-terminal TH helices, exposing hydrophobic sites while retaining a global compact structure. However, the two independent recorded trajectories of 6.8 and 9.5 μs showed differences in the conformational changes observed for these TH helices. Furthermore, coarse-grained MD simulations of T domain in the presence of lipids [39] revealed highly varied membrane insertion modes of the T domain that depended on the trajectory replicas.

The initial models of the protein conformations and protein-membrane association [39] were further used as starting points of microsecond MD trajectories [40], in which the membrane-bound conformations stayed mainly close to the initial conformation, but inserted deeply in the membrane. Extensive equilibrium MD simulations of the domain T at low pH with a combined length of over 8 μs [41] demonstrate that histidine protonation results in substantial molecular rearrangements characterized by the unfolding of helices TH1 and TH2 and the loss of close contacts between the C- and N-terminal segments. During the 6-μs simulation at low pH, the kinking of helix TH1, initiated by rotation of backbone dihedral angles of K216, decreases the α-helical content on the C-terminus of TH1 and was followed by unfolding of its N-terminus. The structural changes include exposure of the TH8-9 hairpin containing the dagger tip, in preparation for its subsequent transmembrane insertion. In addition, pKa of histidines were calculated by Thermodynamic Integration and validated by fluorescence and fluorescence resonance energy transfer (FRET) experiments [41]. Thermodynamic Integration indicates diverse roles for the different histidines in the conformational switching of the T domain triggered by the pH: H223 is a likely candidate for early protonation, but H257 has the highest free energy of protonation, in relation with a large perturbation of the native structure.

The overall view of DT translocation through the endosome membrane is that this process follows a very complex mechanism. The numerous experiments performed on this system have provided some insights, but several contradicting models have been proposed. MD simulations provide a conformational view with respect to the experimental observations, highlighting the great heterogeneity of conformation observed for DT in the different environments studied. The role of some His residues was clarified, but, due to the enormous complexity of the conformational landscape of DT during the translocation, up to now only a superficial exploration was possible, despite the large computational time invested.

### 2.2. Botulinium and Tetanus Toxins

The botulinum toxins (BoNTs) and the tetanus toxin (TeNT) are the most potent toxins known, they target the nervous system and are consequently named neurotoxins [42]. The BoNTs are produced by a variety of anaerobic spore–forming Clostridial species categorized as *Clostridium botulinum*, and cause botulism mostly through food poisoning. Among the BoNTs, various types (from A to G) exist, from which the serotypes A, B and E are lethal. The typical symptom of botulism is flaccid paralysis, which is the inability to contract skeletal muscles, inducing impaired vision, followed by paralysis of facial muscles and ultimately respiratory failure due to diaphragm paralysis. The TeNT is produced by *Clostridium tetani* and causes the disease tetanus, which is drastically different from botulism and is characterized by periodic hypercontraction of skeletal muscles called spastic paralysis.

In agreement with the different symptoms associated to the poisoning, BoNT and TeNT target neuromuscular junction but with distinct destinations, as the catalytic domain of BoNTs is released into the cytosol of motor neurons, whereas the catalytic domain of TeNT is finally released into the cytosol of inhibitory neurons. Both toxin families enter into the synaptic recycling vesicle system, through binding to gangliosides (a class of glycosphingolipids) and to synaptic vesicle membrane proteins. Once the neurotoxin is embarked into a synaptic vesicle, a change of pH toward acidic values induces a conformational change of the toxin, and consequently the translocation of the catalytic domain through the vesicle membrane to the synapse cytosol. This catalytic domain is then separated from BoNT or TeNT and expelled from the vesicle by the cleavage of a very conserved disulfide bridge. The catalytic domain, a zinc-dependent protease, cleaves various SNARE proteins depending on the type of the toxin, thus generating the toxic effect.

**Figure 2:**
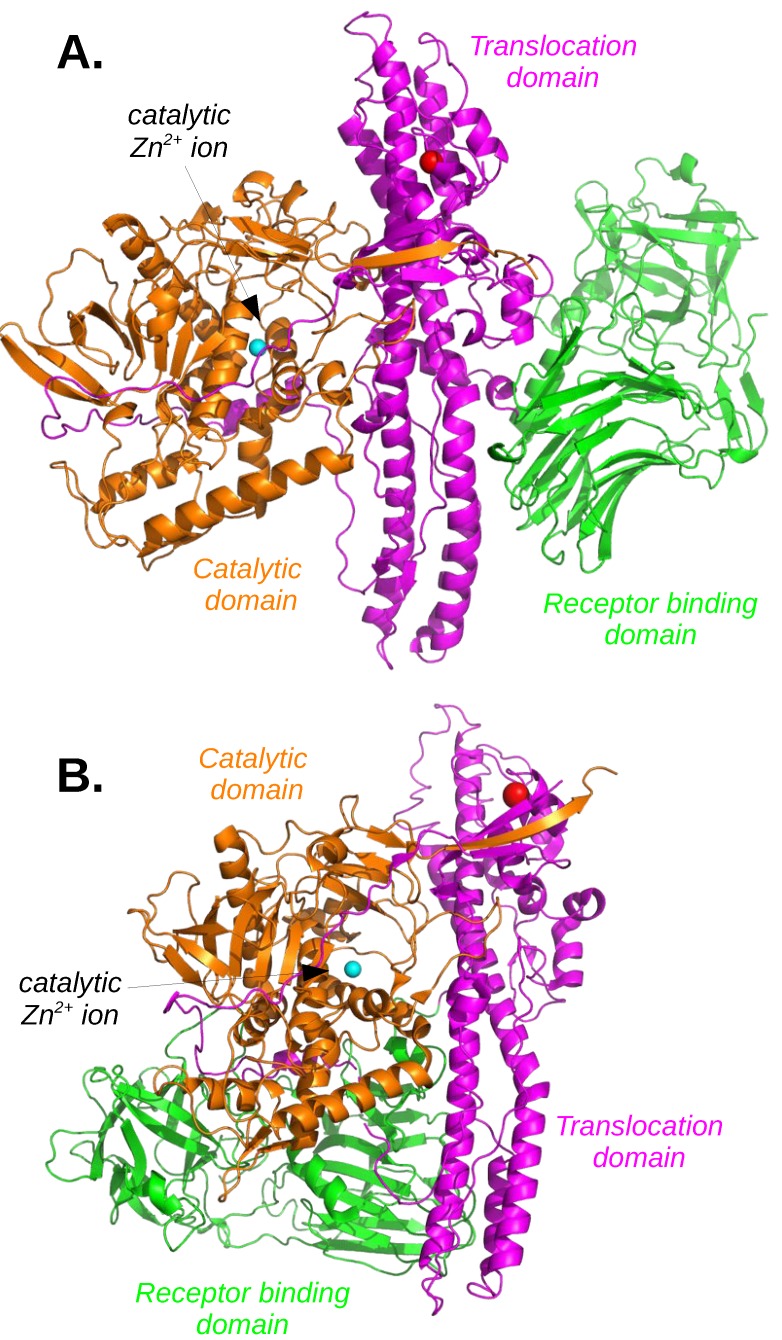
BoNT structures in open (**A**) and closed (**B**) conformations, corresponding to PDB entries: 3BTA (BoNT/A1 [54]) and 3FFZ (BoNT/E [56]). The protein chains are drawn in cartoon, with the translocation domain colored in magenta, the receptor binding domain colored in green, and the catalytic domain colored in orange. The catalytic ion Zn${}^{2+}$ is drawn as a sphere and colored in cyan and an ion Ca${}^{2+}$ (**A**) or Na${}^{+}$ (**B**) located at equivalent positions in the two structures is colored in blue.

BoNTs and TeNT target different neurons, and medium identity percentage in the 30–40% range, is observed for the primary sequences among TeNT and the different types of BoNTs. Nevertheless, all structures determined for these toxins share quite similar organization. At the time of toxin synthesis in *Clostridium botulinum* and *Clostridium tetani*, a unique chain is present. Depending on the toxin types, this chain is cleaved in two peptidic chains, either during secretion from the bacterial pathogen or later within the host. The mature toxins are thus formed by two protein chains (light chain: Lc and heavy chain: Hc) covalently connected through a disulfide bridge. All structures (Figure 2) are organized as a butterfly, the central domain (the butterfly abdomen), named H${}_{N}$, is α helical and corresponds to the translocation domain, helping the catalytic domain to pass the vesicule membrane. One butterfly wing is formed by the light chain previously described, and corresponds to the catalytic domain, a zinc-dependent protease named LC. The other wing (Hc), covalently connected to the H${}_{N}$ domain, contains the N-terminal (H${}_{CN}$) and C-terminal (H${}_{CC}$) receptor-binding regions. These regions, displaying closed β structures similar to immunoglobulin fold, establish the previously described interactions of BoNT with synaptic vesicle membrane proteins and gangliosides.

Several approaches are under development for preventing the lethality of BoNTs. The AntiBotABE program has started the development of an oligoclonal antibody cocktail [43,44,45]. Virtual screening of BoNTs target Lc producing numerous compounds preventing the cleavage of SNARE proteins by binding to the active site of the zinc metalloprotease [46,47,48,49,50,51,52,53]. But, by contrast and thanks to their characteristic paralysis effects, BoNTs are also used to treat an increasing number of medical disorders [42] including human neuromuscular disorders characterized by involuntary muscle contractions (strabismus, blepharospasm, and hemifacial spasm), as well as cosmetic applications.

In addition of displaying similar structure organization, X-ray crystallographic structures revealed relative similar positions of domains among BoNTs and between BoNTs and TeNT (Figure 2). Several BoNT types (BoNT/A1 [54], BoNT/B1 [55]) are in an open conformation, displaying a flat position of the wings, as for a naturalized butterfly (Figure 2A). Whereas, the BoNT/E [56] and TeNT [57] structures display two distinct closed conformations, in which both wings are folded together, as if the butterfly was perched upon a flower (Figure 2B), with relative different positions of the wings between the two toxins. Another intermediate conformation of BoNT is observed for progenitor toxin complexes (PTC) of BoNT/A1 [58], and BoNT/E [59], which protects the toxin and facilitates its absorption in the gastrointestinal tract. In all structures, a very long loop, the belt, belonging to the chain Hc, grips the catalytic domain LC. Two Ca${}^{2+}$, described as essential [60] for the translocation, are bound within the translocation domain of BoNT/B1 [60]. Interestingly, in the BoNT/E structure [56], a Na${}^{+}$ ion is located at a position equivalent to that of a Ca${}^{2+}$.

Another important aspect of the conformational landscape of BoNTs is the influence of the pH. As described above, acidic pH in synaptic vesicles induces a conformational change of BoNTs. As pH drops under 6, variation of protonation in histidine residues could play a major role in this change. Nevertheless, X-ray crystallographic structures of BoNT/B, determined at various pH values [60], do not display any variation of conformation. But, decrease of pH was also shown [61] to induce a decrease in ellipticity of the CD spectra. Small angle X-ray scattering (SAXS) measurements on TeNT revealed a conformational change as a function of pH [57]. The formation of the BoNT/E PTC complex is similarly favored by pH variations and networks of acidic/basic residues were put in evidence at the interface between BoNT/E and the non-toxic-non-hemagglutinin protein (NTNHE) [59].

The conformational landscape of BoNTs presents several interesting aspects. First, it is a fascinating example of large conformational variations in close relationship with important physiological processes. Second, shifts in pH play an important role in inducing structural changes, and it is challenging to properly model such effects. Third, understanding the mechanism of action of BoNTs will open the way to educated engineering of these toxins. Despite this, only one MD simulation study [62] have been performed [62] on an BoNT/A uncleaved chain.

DT and BoNT/TeNT toxins have numerous similar features in common. These toxins are composed of two polypeptidic chains attached by a disulfide bridge. Their structures display packed/extended and open/closed conformations, as well as non toxic dimers stabilized through a network of hydrogen bonds between acidic residues. The R domain of DT displays an immunoglobulin fold which is also encountered in the domains HCC and HNN of BoNTs and TeNT. The translocation domain in both proteins is formed from α helices and the catalytic domains display an α/β fold.

However, these two protein families display also strong differences: (i) their size, about 500 residues for DT, about 1300 residues for BoNT and TeNT; (ii) a quite intricate topology for BoNT/TeNT with a belt region extending from the HN domain to grip the catalytic LC domain, whereas the topology of DT is based on more short-range interactions. The BoNT/E structure [56] displays disordered regions, whereas a more limited disorder is observed in any DT structure [17].

## 3. Adenylyl Cyclase Virulence Factors

The adenylyl cyclase toxins are present in several pathogens. Up to now, three of these toxins have been studied at the molecular level: the edema factor (EF) from *Bacillus anthracis*, CyaA from *Bordetella pertussis* and ExoY from *Pseudomonas aeruginosa* [63,64]. X-ray crystallographic structures have been determined for EF [65], CyaA [66] and ExoY [67]. EF and CyaA are activated as adenylyl cyclase by interaction with the ubiquitous protein calmodulin (CaM), present in the cytoplasm of the host cell. The activated adenylyl cyclase triggers overproduction of cyclic adenosine monophosphate (cAMP), which in high concentration perturbs the cell signaling system making its immune response inefficient. ExoY is activated by interaction with filamentous actin [64]. Results obtained by molecular modeling on the catalytic domains of CyaA and EF have been reviewed in details in Refs. [68,69].

### 3.1. Plasticity of the Adenylyl Cyclase Interaction with Calmodulin

**Figure 3:**
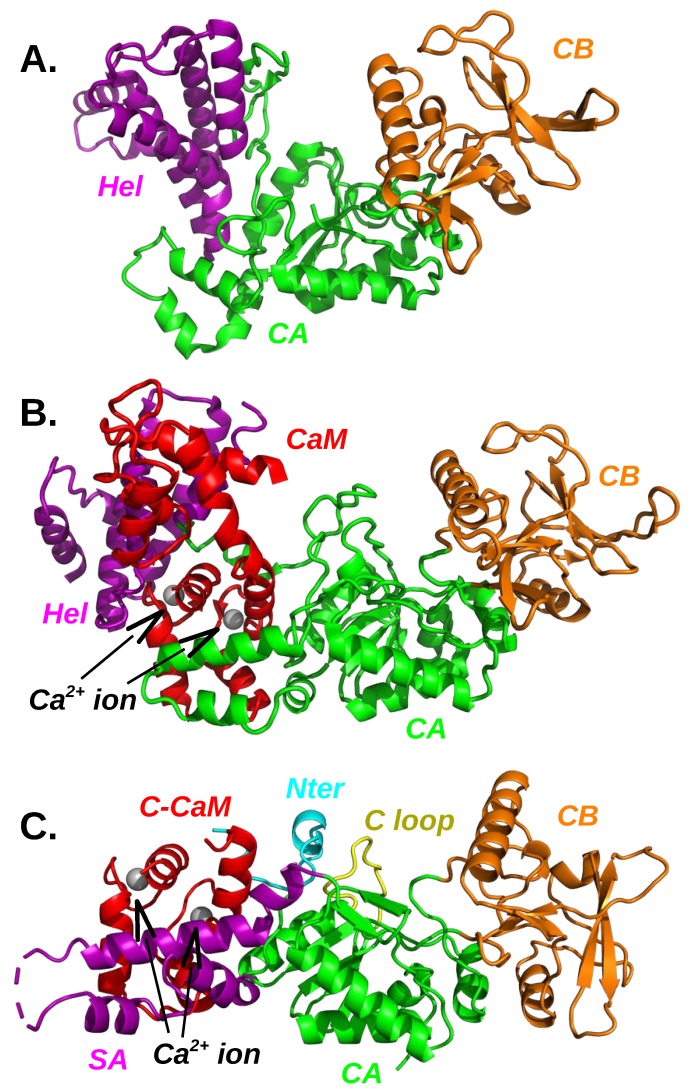
Adenylyl cyclase structures: (**A**) isolated EF (PDB entry: 1K8T [65]), (**B**) EF/CaM complex (PDB entry:1K90 [65]), (**C**) AC/C-CaM complex (PDB entry: 1YRT [66]). The full calmodulin (CaM) and the C terminal lobe of calmodulin (C-CaM) are colored in red with ions Ca${}^{2+}$ in silver. The domains CA and CB are colored in green and orange. The helical domain (EF) and the SA domain (AC) are colored in magenta. In the AC/C-CaM complex (C), the N terminal tail (Nter) of AC is colored in cyan and the catalytic loop (C loop) is colored in yellow.

CyaA revealed itself as a very good prototype for developing various biotechnological applications [70,71] which then drove the interest for its structure [66]. On the other hand, the interest in edema factor is based upon defence against its potential to cause harm in anthrax-based bio-terrorism [72]. The availability of high-resolution X-ray crystallographic structures for free EF, EF complexed to CaM and the catalytic domain AC of CyaA complexed to the C terminal lobe of CaM (C-CaM) (Figure 3) permits to study the adenylyl cyclase dynamical properties and to relate them to the toxin function. For the AC/CaM complex, the N-terminal lobe of CaM has been predicted to bind AC at different positions [73]. The enzymatic reaction of anthrax adenylyl cyclase has also been examined through the determination of various X-ray crystallographic structures including the reactant [74] and the reaction products [75] and showing EF uses two-metal–ion catalysis [76,77,78].

The interactions of AC and EF with calmodulin are different, although they share similar global features (Figure 3). The barrier of activation of AC is smaller than the one of EF, as the affinity of AC for calmodulin is about 0.2 nM [79], whereas it is 20 nM for EF [66,80]. This difference is also visible in an analysis of energetic influences. This coarse-grained analysis model [81,82], obtained by dividing the two partners of interaction into regions, analyzes the energetic influences between these regions. The influence diagrams obtained for the EF/CaM and AC/C-CaM complexes are qualitatively different as many more influences and a more intricate pattern are observed for the EF/CaM than for the AC/C-CaM diagram. This topological feature is not surprising, as the interaction between EF and CaM required to move apart the EF helical domain and the CA domain in order to insert CaM (Figure 3A,B). In contrast, the α helix H of AC, to which C-CaM is in direct interaction (Figure 3C), is more accessible. Consequently, the surface of interaction between adenylyl cyclase and CaM is larger for EF/C-CaM than for AC/C-CaM.

Observation of AC/CaM interaction in the X-ray crystallographic structure [66] (Figure 3C) suggests that this interaction mostly arises from the α helix H of AC. Indeed, mutations of CaM methionines, belonging to the CaM hydrophobic patch [83], strongly decrease the AC affinity for CaM [84]. Furthermore, the α helix H can be considered as a structural anchor, because the peptide spanning the sequence of helix H folds as an α-helix in solution [85,86].

However, available MD trajectories allowed to enlarge this point of view. Indeed, the removal of Ca${}^{2+}$ from the complex AC/C-CaM induced during the course of MD simulations [82] the breaking of hydrogen bonds involving the AC residues D360, R338 and N347 located in the C-terminal extremity of AC, the AC residues Q302, E301 and N304 located in the catalytic loop, and the C-CaM residue R90 located in an α helix of the EF hand 3. The hydrogen bonds connecting these residues form a network from C-CaM to the AC catalytic site and their breaking is thus directly related to the AC function. The importance of these residues was confirmed by mutagenesis studies [87]: mutation to alanines induces a decrease of the affinity of AC for CaM, which agrees with the model of network described above and with a more complex interaction interface than the one initially guessed. Independent experimental studies of CaM [88] reveal that the interaction of calmodulin with AC increases the apparent Ca${}^{2+}$ binding in C-CaM.

In the complex AC/C-CaM, the loop containing residues 226–232, located at the extremity of SA region which is not visible in the crystal structure, but has been reconstructed using Modeller [89], displays large internal mobility along the MD trajectories [82]. The other parts of the SA region (Figure 3C) display larger mobility if Ca${}^{2+}$ are removed, and even more in the free protein AC. The SA mobility is induced by large variations of relative orientations between α helices. Using a combination of SAXS, HDX-MS, and synchrotron radiation circular dichroism (SR-CD), it was shown [90] that, in the absence of CaM, AC exhibits significant structural disorder, and that a 75-residue-long stretch within AC undergoes a disorder-to-order transition upon CaM binding.

In the structures of AC, the C terminal lobe of CaM is complexed with two Ca${}^{2+}$. By contrast, several levels of complexation are present in EF/CaM complexes. An NMR study showed that the calcic loops of the C terminal lobe of CaM display the best affinity for Ca${}^{2+}$ ions [91]. For MD trajectories recorded on EF/CaM complexes using various levels of complexation by Ca${}^{2+}$ [92], CaM displays tendency to be more or less elongated and is the best fitted to the interaction with EF, in the case of two Ca${}^{2+}$ bound to C-CaM. The MD simulations of AC/CaM [82] and EF/CaM [81,92,93], revealed that unlike observations on isolated calmodulin [94,95], the open conformation of the helix-turn-helix motifs (the so-called EF hand) is kept in the presence as well as in the absence of Ca${}^{2+}$.

Along the course of MD simulations [82], free AC conformations show a general tendency to become less elongated, by compacting the protein’s extremities, the regions SA and CB. Nevertheless, the conformational drift of AC corresponds to oscillations around the X-ray crystallographic structure [66] and no conformational transition to a new basin was observed for the protein [82]. Enhanced sampling approaches, as temperature accelerated MD (TAMD) [96,97] and sr-TAMD [98], were used to further explore the conformational landscape of AC. The sr-TAMD approach allowed to obtain series of AC conformations [98] displaying a significantly less elongated shape than the starting X-ray crystallographic structure, as well as the conformations sampled in the previous TAMD. This decrease in elongation is obtained by a large reorganization of the α helices in the SA domain. The β hairpin (residues 259–273) is in most conformations less accessible to the solvent than in the AC/CaM complex, in agreement with its important role in the interaction with the N-terminal lobe of CaM (N-CaM) [99].

### 3.2. Searching Inhibitors for Adenylyl Cyclases

Whooping cough, caused by *Bordetella pertussis*, remains predominantly sensitive to antibiotic treatment, yet resistant strains are evolving [100,101] making the search for toxin inhibitors a sensible precautionary measure [100,102,103,104,105,106]. Search for inhibitors of edema factor have been also performed [102,107,108,109,110,111,112,113,114] and are reviewed in [113]. Some compounds are active against both CyaA and EF [115,116]. Monoclonal antibodies were raised against EF [117].

A virtual screening study of the adenylyl cyclase EF from *Bacillus anthracis* led [118] to the discovery of EF inhibitors, belonging to the series of thiophen ureidoacids. This study targeted the pocket SABC, located between the catalytic loop and the CaM binding site, which displays a large variation of shape between the active and inactive states of EF. Surprisingly, these EF inhibitors also displayed activity against AC [118], which was discovered by chance when AC was used as a control to avoid the detection of promiscuous ligands. The similarity between the inhibition of EF and AC is supported by the sequence alignment of proteins of the multifunctional-autoprocessing repeats-in-toxin (MARTX) toxin family [119], including EF, AC and ExoY. Although the sequence similarity is not very high, the alignment coupled to the possible similarity of the inhibitors interaction in EF and AC can be exploited to push forward the structural, biophysical and biochemical knowledge on the proteins MARTX.

## 4. Conclusions

The examples described here illustrate the importance of the combined use of molecular modeling and experimental structural biology approaches. Indeed, from the initially determined structures of EF and AC, it would have been impossible to guess that the same family of compounds, the thiophen ureidoacids, could inhibit the enzymatic activity of both toxins. The use of molecular modeling can thus make possible to rationalize structural properties. Similarly, the comparison of the DT and BoNT/TeNT structures enabled detecting common physico-chemical properties, such as the open/closed conformation transition or the partial unfolding at low pH. Therefore, it could be expected that the description of the DT already obtained is qualitatively transferable to toxins BoNT and TeNT.
